# Coverage and predictors of the uptake of the mass drug administration of praziquantel chemotherapy for schistosomiasis in a selected urban setting in Zambia

**DOI:** 10.3389/fepid.2023.1168282

**Published:** 2023-04-24

**Authors:** Felix Nzonzi Kiesolo, Mutale Sampa, Given Moonga, Charles Michelo, Choolwe Jacobs

**Affiliations:** ^1^ Department of Epidemiology and Biostatistics, School of Public Health, University of Zambia, Lusaka, Zambia; ^2^ Strategic Centre for Health Systems Metrics & Evaluation, School of Public Health, University of Zambia, Lusaka, Zambia; ^3^ Harvest Research Institutes, Harvest University, Lusaka, Zambia; ^4^ Women in Global Health, Lusaka, Zambia

**Keywords:** schistosomiasis, mass drug administration, uptake, praziquantel chemotherapy, intervention coverage, bilharzia

## Abstract

The burden of schistosomiasis in Zambia has remained high over the years. The World Health Assembly recommended adequate mass drug administration coverage for schistosomiasis using Praziquantel chemotherapy for school-aged children and all at-risks adults. We aimed at investigating the coverage and the factors associated to the uptake for MDA for schistosomiasis in Ng'ombe township of Lusaka, Zambia. A cross-sectional survey was conducted in May and June 2021 *via* phone calls to the residents of Ng'ombe township. Commcare software was used in the conduct of the survey. Pearson's Chi-square test and multiple logistic regression were conducted using the STATA version 15.0. 769 study participants were randomly selected using systematic sampling, of which 76.3% were younger than 40 years, 64.9% were female, 64.4% were married, 56.3% had reached the secondary educational level and 51.9% were employed. Coverage for MDA for schistosomiasis in Ng'ombe township in 2018 was found to be 49.8% (95% CI: 46.2%–53.4%). Positive predictors of the MDA were prior knowledge of the occurrence of the MDA in 2018 (aOR: 2.892, *p* < 0.001) and believing that the provision of incentives like snacks was important during the MDA with PZQ in Ng'ombe township (aOR: 1.926, *p* = 0.001), whereas age (aOR:0.979, *p* = 0.009), marital status (aOR:0.620, *p* = 0.006), employment status (aOR:0.587, *p* = 0.001) were negative predictors of the MDA. Elimination of the burden of schistosomiasis in endemic settings needs the attainment of an optimum coverage and uptake during MDA with PZQ. Therefore, prior knowledge about an impending intervention and the provision of incentives like snacks during the intervention should be prioritized by MDA implementers, while background characteristics such as age, marital status, and employment status need to be taken into consideration when planning and promoting uptake in future MDAs.

## Introduction

Schistosomiasis remains a neglected tropical disease across the world (1). Globally, it has been estimated that 779 million people are at risk of schistosomiasis, and 250 million people are infected by the illness (2, 3). Furthermore, schistosomiasis accounts for an estimated 3,514,145 disability-adjusted life years (DALYs) (4). The disease has two main symptomatologic presentations: the hepatointestinal presentation and the urogenital presentation (5, 6). Hepatointestinal schistosomiasis can present either as hepatic schistosomiasis, which manifests as liver fibrosis or portal hypertension, or as intestinal schistosomiasis, which is associated with symptoms such as headache, diarrhea, fever, loose stools, stomach ache, and malnutrition (7, 8). Urogenital schistosomiasis, on the other hand, can be divided into two forms: urinary schistosomiasis, which is mainly associated with an altered urination frequency, difficulty urinating, or hematuria (9), and genital schistosomiasis, which can be further divided into male genital schistosomiasis (MGS) and female genital schistosomiasis (FGS), as they refer to the genital manifestation of the illness in males and females, respectively (10, 11). Although mostly underreported, the chronic form of the disease can cause death directly or indirectly (12).

Schistosomiasis is endemic in Sub-Saharan Africa (SSA), where it mainly affects the poor and the underprivileged (3, 13, 14). The disease has significant economic and public health implications in developing countries where it mostly affects populations, particularly in rural and some peri urban areas. The illness is endemic in Zambia, with a widespread distribution throughout the country, and affects 4 million school-aged children in a total population of 18 million (15, 16).

In 2001, the World Health Assembly (WHA) recommended that an annual and periodic mass drug administration (MDA) with Praziquantel (PZQ) should cover at least 75% of school-aged children (SAC) to address the global problem of human schistosomiasis in endemic settings (17). In 2020, the World Health Organization (WHO) (18) published a new roadmap to help progress the control and elimination of neglected tropical diseases (NTD) by 2030. The WHO recommended the elimination of schistosomiasis as a public health problem and ending the transmission of schistosomes in human hosts in selected countries by 2030 by considering the WASH strategy (safe drinking-WAter, Sanitation infrastructure and Hygiene education), the control of snail populations, and the preventive chemotherapy (2020). Preventive chemotherapy with PZQ remains an effective approach for combatting the transmission of schistosomes in humans and has to be administered annually in endemic settings.

The government of the Republic of Zambia (GRZ) adopted the WHA recommendation. Through the national health master plan, the country set strategies for the elimination of NTDs by 2023. These included scaling up MDA campaigns, integrating NTD control activities into the primary health care services, and formulating health promotion programs (sanitation and hygiene) aimed at preventing and reducing NTDs. According to the Ministry of Health (MoH), MDA campaigns using praziquantel is primarily carried out in schools as SAC of 5–14 years are the target population. In communities with a prevalence above 50%, at-risk adolescents and adults are also treated through a community-based approach or door-to-door administration of drugs (15).

Despite the inexpensive and effective MDA of Praziquantel chemotherapy, the burden of schistosomiasis continues to be of concern in the country. Kalinda et al. argue that “the prevalence of schistosomiasis continues to be high, especially among poor and marginalized communities” (2018). Despite limited schistosomiasis prevalence studies conducted in Zambia over the years, an attempt to estimate the prevalence of the illness was made through a systematic review and meta-analysis, which yielded pooled prevalence estimates of 34.9% and 35.5% for *Schistosoma mansoni* and *Schistosoma haematobium*, respectively (16)**.**

Furthermore, a longitudinal cohort study conducted in Ng'ombe township in Lusaka district from 2007 to 2015 showed that pre-MDA schistosomiasis prevalence was 28.6% [95% confidence interval (95% CI): 25.8–31.5] among primary school children in 2007. Following the implementation of the MDA with PZQ in Ng'ombe township in 2010, the prevalence of the illness decreased to 20.3% (95% CI: 15.8–24.8) in 2011, after which the prevalence increased to 38% (95% CI: 32.9–43.1) in 2015 (19).

In 2018, the Zambian Ministry of Health implemented another mass treatment with PZQ in the township in a bid to reduce the prevalence and burden of the illness. However, evidence with regard to the coverage and predictors of the uptake of the MDA with PZQ chemotherapy in Lusaka is limited. In this study, we examined the coverage and the factors associated with the uptake of MDA with PZQ in Ng'ombe township in Lusaka, Zambia.

## Methods

### Study design and setting

This was a cross-sectional study conducted from April 2021 to May 2021 in Ng'ombe township, a peri-urban slum area located in the Lusaka district of Zambia. Ng’ombe township has approximately 95,000 inhabitants and consists of homes that are built too closely to one another, without any proper road matrix, water supply, or electricity. Ng’ombe township was purposively selected due to the disease burden among its residents than in other townships in Lusaka.

The consolidated framework for implementation research (CFIR) was used to conceptualize factors associated with the uptake of MDA with PZQ. The CFIR framework facilitates an integral analysis of the factors that interplay and intertwine in the implementation of an intervention. It encompasses five domains: the intervention, the outer setting, the inner setting, the individual characteristics, and the implementation process (20). In the context of the present study, the individual characteristics and the implementation process of the CFIR framework facilitated the selection of the independent variables. The CFIR also guided the discussion of the results.

### Study participants

The study population was the population living in Ng'ombe township. Therefore, a study participant was included in the present research if they were aged between 18 and 69 years of age, lived in Ng'ombe township during the last MDA with PZQ in 2018, and had a phone number through which they could be contacted. Any potential participant living in Ng'ombe township but who had traveled or was not physically present in the township during MDA with PZQ for schistosomiasis in 2018 was not included in the present study.

### Survey development

A review of scientific articles on the topic of the uptake of MDA for schistosomiasis was conducted, and the questionnaires that were used on the population where the MDA for schistosomiasis took place were used in the context of the present study. Therefore, the implementation outcome variable was the *uptake of the MDA with PZQ* (or having swallowed a PZQ tablet during the MDA). Independent variables were age, gender, marital status, employment status, education level, and years lived in Ng'ombe. Other independent variables included having heard of schistosomiasis, prior knowledge of the occurrence of the MDA, having received a PZQ tablet during the MDA in 2018, and the use of incentives, such as snacks, during the MDA. Following the development of the questionnaires, they were tested and later administered by trained research assistants during the survey.

### Sampling procedures and sample size

For the sampling strategy, one-stage cluster sampling was used. At this stage, a computer-led random sampling of 11 health zones within the Ng'ombe community was conducted. Within each selected health zone, the phone numbers of individuals were collected to form the sampling frame for the systematic random sampling of the participants included in the study.

The starting point was the house of the community leader. The phone numbers were collected by trained community health workers in Ng'ombe township, as they were given a daily target of phone numbers to collect. This collection was carried out over a period of 4 months from January 2021 to April 2021. During this process, the measures intended to prevent the spread or contamination of the coronavirus were observed.

In total, 14,000 phone numbers were collected and 1,497 were discarded as they contained errors or were from health zones not previously selected. At this point, 12,503 phone numbers formed the sampling frame from which 769 phone numbers were randomly selected using a systematic sampling approach in which the kth number was 16. Therefore, 769 study participants were included in the present study.

The determination of the sample size was carried out using the prevalence formula. Considering the prevalence of 50%, the z*_α_*_/2_ corresponding to the 95% confidence interval of 1.96, and a margin of error of 5%, the minimum sample size (n) was estimated at 384.16.

### Data management and analysis

Data were collected using structured pretested questionnaires. The questionnaires were administered to the study participants through phone calls with research assistants fluent in English, Nyanja, and Bemba. Data were collected between April 2021 and May 2021, for approximately 1 month.

For each participant, a distinct identity code was developed. The data collectors were trained in survey methodology to ensure data validity. The lead investigator checked for missing values before their export from Commcare software into an Excel spreadsheet. To test for reliability, a visual check and normality plots were used. Thereafter, the data were imported into STATA software, version 15.0 SE (Stata Corporation, College Station, TX, USA), for the analysis.

To test the association between the uptake of the MDA for PZQ and the independent variables, the Pearson's Chi-square test and corresponding *p*-values were calculated. Afterwards, an investigator-led logistic regression model was used for the survey data.

Finally, the model specification was used for the logistic regression diagnostics. This helped to choose the correctly specified model between the full model and the investigator-led logistic regression final model (or nested model). A properly specified model suggests that relevant variables have not been omitted. On the other hand, a model that is not properly specified implies that relevant variables have been excluded. The linktest command was used for model specification.

### Ethical considerations

This study was approved by the University of Zambia Biomedical Research Ethics Committee (UNZABREC) (REF. No. 1105-2020) and the National Health Research Authority (NHRA) (Ref No: NHRA00001/16/11/2020). Moreover, all study participants verbally consented to freely participate during the survey in the present study after they were informed about the risks. Furthermore, the participants’ phone numbers were kept confidential during and after the survey.

## Results

### Socio-Demographic characteristics of the study participants

Table 1 summarizes the background of the participants. Overall (*n* = 769), 64.9% were females and 35.1% were males. The participants median age was 32 (IQR: 26, 39) and the majority (76.3%) were younger than 40 years. Furthermore, 64.4% were married, 56.3% had reached secondary educational level and 51.9% were employed. The median year spent in the Ng'ombe township by the study participants was 14 (IQR: 8, 21).

**Table 1:** Background characteristics of the participants.

Variables	Descriptive statistics	
Continuous	Median	Interquartile range
Age	32	(26, 39)
Years spent in Ng’ombe	14	(8, 21)
Categorical	Frequencies	Percentages
**Sex**		
Male	240	35.1%
Female	499	64.9%
**Marital status**		
Not married	274	35.6%
Married	495	64.4%
**Education level**		
Primary level	218	28.4%
Secondary level	433	56.3%
Tertiary level	118	15.3%
**Employment status**		
Not employed	369	48.1%
Employed	399	51.9%

### Coverage of the mass drug administration for schistosomiasis in Ng'ombe township

Coverage of the MDA with Praziquantel chemotherapy for schistosomiasis in Ng'ombe township was 49.8% (95% CI: 46.2–53.4%) in 2018.

### Investigating the predictors of the uptake of MDA using logistic regression

The predictors of the MDA with PZQ for schistosomiasis in Ng'ombe township in 2018 are shown in Table 2 after conducting a stepwise backward elimination process.

**Table 2:** Non-adjusted and adjusted logistic regression estimates of the uptake of the MDA against each potential predictor variable.

Factor	Proportion	Crude Odds ratio (95% Confidence interval)	*p*-value	Adjusted Odds ratio (95% Confidence interval)	*p*-value
Age	–	0.961 (0.948–0.975)	**<0.001**	0.979 (0.963–0.995)	**0.010**
**Sex**					
Male	0.35	1(ref)			
Female	0.65	1.459 (1.092–1.948)	**0.011**	—	–
**Marital status**					
Unmarried	0.36	1(ref)		1(ref)	** **
Married	0.64	0.473 (0.353–0.634)	**<0.001**	0.620 (0.438–0.878)	**0.007**
**Education level**					
Primary	0.28	1(ref)			
Secondary	0.56	1.313 (0.956–1.802)	0.092		
Tertiary	0.15	0.713 (0.458–1.110)	0.134	—	–
**Employment status**					
Unemployed	0.48	1(ref)		1(ref)	
Employed	0.52	0.473 (0.331–0.580)	**<0.001**	0.587 (0.428–0.804)	**0.001**
Years lived in Ng’ombe township	–	0.99 (0.976–1.004)	0.145	—	–
**Hearing of MDA before 2018**					
No	0.02	1(ref)	* *		
Yes	0.98	2.641 (0.961–7.261)	0.060	—	–
**Delivery mode**					
Central location	0.637	1(Ref)			
Door-to-door	0.131	1.279 (0.882–1.856)	0.194	—	–
**Prior knowledge of the occurrence of the MDA**					
No	0.32	1(ref)	** **	1(ref)	** **
Yes	0.68	3.061 (2.240–4.186)	**<0.001**	2.892 (2.071–4.038)	**<0.001**
**Use of incentives like snacks during the MDA**					
No	0.21	1(ref)	** **	1(ref)	** **
Yes	0.79	2.418 (1.692–3.457)	**<0.001**	1.926 (1.302––2.850)	**0.001**

A univariable logistic regression revealed that the odds of the uptake of the MDA with Praziquantel reduced by 4% with age (*p* < 0.001, 95% CI: 0.948–0.975), increased by 45% for females (*p* = 0.011, 95% CI: 1.092–1.948), decreased by 52.7% for married people (*p* < 0.001, 0.353–0.634), and decreased by 56.2% for people in employment (*p* < 0.001, 0.331–0.580). Additionally, the odds of swallowing a PZQ tablet during the MDA in 2018 was 2.641 times more likely if one had heard of the MDA before 2018 (*p* = 0.060, 0.961–7.261). Furthermore, prior knowledge of the occurrence of the MDA in Ng'ombe township in 2018 increased the odds of swallowing a PZQ tablet during the MDA 3.061 times (*p* < 0.001, 2.240–4.186), and the provision of incentives, such as snacks, during the MDA would have increased the odds of swallowing a PZQ tablet during the MDA 2.418 times (*p* < 0.001, 1.692–3.457).

Moreover, after conducting a multiple logistic regression, taking the other variables in the model into account, the final investigator-led model reveals that a 1-year increase in age decreased the odds of swallowing a PZQ tablet during the MDA by 2% (*p* = 0.01, 95% CI: 0.963–0.995), that being married decreased the odds of swallowing a PZQ tablet during the MDA by 40% (*p* = 0.007, 95% CI = 0.438–0.878), and that being employed decreased the odds of swallowing a PZQ tablet by 42% (*p* = 0.001, 95% CI: 0.428–0.804).

Moreover, taking account of the other variables in the model, the odds of swallowing a PZQ tablet for those who had prior knowledge of the occurrence of the MDA were increased 2.892 times (*p* < 0.001, 2.071–4.037), and the odds of the uptake of the MDA increased by 92% if incentives such as snacks were provided during the MDA (*p* = 0.001, 1.302–2.850).

### Conducting the logistic regression diagnostics using the model specification

The model specification of the two competing models is shown in Tables 3, 4. The significant *p*-value of the linear predicted value squared (hatsq) in the full model suggests that the linktest is significant, and therefore, the full model is not properly specified, suggesting that relevant variables in the full model have been omitted. The insignificant hatsq of the nested model suggests that the linktest is insignificant. Therefore, the nested model is properly specified, suggesting that no relevant variables were omitted.

**Table 3:** Model specification of the full model.

Uptake of the MDA of Praziquantel	*t*	*p*-value	95% C.I.
hat	9.78	<0.0001	0.775–1.163
hatsq	−2.20	0.028	(−0.380)–(−0.022)
cons.	1.29	0.198	(−0.066)–(0.319)

**Table 4:** Model specification of the nested model.

Uptake of the MDA of Praziquantel	*t*	*p*-value	95% C.I.
hat	9.92	<0.0001	0.807–1.205
hatsq	0.34	0.731	(−0.140)–(0.200)
cons.	−0.18	0.860	(−0.202)–(0.169)

## Discussion

The present study revealed that coverage of the MDA conducted in Ng'ombe township was 49.8% in 2018. Negative predictors of the uptake of the MDA were age, being married, and being employed, while prior knowledge of the occurrence of the MDA and the use of incentives such as snacks during the MDA were positive predictors of the uptake of the MDA.

The results are comparable to the findings of a mixed methods cross-sectional study in Central Uganda, where the coverage was below the 75% treatment coverage recommended by the WHO and where prior knowledge of schistosomiasis increased the uptake of the MDA (21). The findings of the present study are also consistent with the SCORE Project conducted by Omedo et al. in western Kenya in that a high uptake of the MDA was almost always the result of a proper communication campaign (2014). The results in this study are also consistent with a cross-sectional study conducted in Uganda in which the coverage was below the coverage threshold recommended by the WHO (22).

### Implementation process of the MDA for schistosomiasis in Ng'ombe township

Coverage of the MDA (49.8%) fell short of the 75% threshold above which the coverage of the intervention would be considered acceptable by the WHO. Possible reasons for that MDA coverage can be explained but may not be limited to the factors pertaining to the intervention recipients, the core product of the intervention itself, and/or factors related to the implementation of the intervention. These factors can overlap each other.

Therefore, 49.8% is consistent with other studies conducted in Uganda and Zanzibar (23–25). As a matter of fact, many studies yielded lower coverages of the MDA with PZQ for schistosomiasis than that reported in the present study (21, 22, 26, 27). Moreover, lower intervention coverage rates have also been noted in different urban settings in other mass treatment studies (28, 29).

The finding that more than half of the adults in Ng'ombe township did not take a PZQ tablet during the MDA with PZQ chemotherapy in 2018 threatens the elimination of schistosomiasis, as argued by Halwindi et al. (30). And yet, reaching all at-risk adults in endemic areas is one of the WHO recommendations for avoiding high infection intensity (31).

However, higher coverage of the MDA in studies conducted in similar settings in Mali, Tanzania, and Uganda proves that a proper and adequate preparation of the implementation of the MDA for schistosomiasis in Ng'ombe township among at-risk adults in the community is feasible (32–34).

### Individual characteristics of the MDA for schistosomiasis in Ng'ombe township

In this study, we found that older people were less likely to take part in the MDA, contrary to the result that was found in a similar study conducted in west Mali where age was found to be positively associated with the uptake of the MDA with PZQ (32). However, a study conducted in Uganda found that age had no effect on the uptake of the MDA for schistosomiasis (23).

In addition, adults were less likely to take part in an MDA for schistosomiasis than SAC, as the SAC were said to be influenced by a guardian or parental figure, whereas adults were more prone to decide independently on whether to participate in a subsequent MDA or not (26, 32, 35). Although adults are more independent decision-makers, a pre-MDA health promotion and sensitization targeting adults would certainly yield positive results in future MDAs with PZQ.

This study also found that being employed was associated with a decreased uptake of the MDA with PZQ in Ng'ombe township. Two studies published in Ivory Coast and Zanzibar found that non participation during the MDA for schistosomiasis was related to work and being busy (25, 36). Similarly, in another study conducted in Uganda by Adriko et al., it was noted that not being present in the community during the MDA for schistosomiasis decreases the odds of participation in it (2018). The possible justification as to why being employed negatively influenced the uptake of the MDA for schistosomiasis may be related to that of age in that young adults enjoyed high employability in the work market, resulting in them being more likely to be at a place of work during an MDA (37, 38). Therefore, the timing of the MDA is an important factor as employed individuals are less likely to be in the community during working hours. Targeting such a group mostly at the weekend is necessary.

The present study found that being married negatively predicted the uptake of the MDA with PZQ in Ng'ombe township in 2018. This finding is contrary to previous studies. However, although the mechanism for this finding is unclear, it was argued by married men in a qualitative study conducted in North-West Uganda that the fertility of women was somewhat compromised by PZQ. Married women were said to have not conceived from the time of the previous MDA with PZQ conducted in 2004 to the time of the interview in 2007. Additionally, women who were pregnant were afraid to take the drug as they were convinced it could lead to a miscarriage or an abortion (39).

While being married was a negative predictor for the uptake of the MDA, married people may obviously fall in the group of individuals who are of an increasing age and more likely to be employed; therefore, the justification for them not swallowing a PZQ tablet during the MDA may as well be the same as that for older and employed people (40, 41).

Prior knowledge of the occurrence of the MDA in 2018 was a positive predictor of the uptake. Therefore, if people were aware of the impending MDA with PZQ, they were more likely to swallow a PZQ tablet (42). This suggests that when people are prepared for it, they present less resistance, unlike when people feel that a PZQ tablet is given to them by surprise without any form of warning. In a cross-sectional survey conducted in the Philippines involving 2,189 adults, it was found that being informed about an impending MDA with PZQ for schistosomiasis significantly reduced non-compliance, thus predicting an increasing uptake (43). In another study conducted in Kenya, it was found that communication campaigns improved compliance (44). These two studies support this study's finding that prior knowledge of the MDA with PZQ for schistosomiasis increased its uptake.

Similarly, the use of incentives, such as snacks, during the MDA with PZQ in Ng'ombe township in 2018 predicted an increase in its uptake. Most recipients of the MDA with PZQ felt more confident to participate in the MDA with PZQ when snacks were provided to them during the MDA to mitigate the side-effects related to PZQ chemotherapy. This was also true when those who were informed in advance of an impending MDA made sure they had a meal prior to taking a PZQ tablet to reduce the side effects associated with PZQ chemotherapy.

Moreover, the use of incentives such as snacks during the MDA is parallel to findings in a cluster randomized trial conducted by Muhumuza et al. in Uganda involving 12 schools where health education was provided but snacks were only provided to six of them during the subsequent MDA. A high uptake and low side-effects related to the PZQ tablet were noted in the schools that were provided snacks (2014). In a separate study conducted in north-western Tanzania, Bukindu F. et al. found that the provision of food was associated with a higher uptake of the MDA with PZQ for schistosomiasis (45). The provision of incentives such as snacks is so critical to the success of an MDA for schistosomiasis in that in the absence of snacks the compliance to the MDA with PZQ dwindles away, as was demonstrated in serial cross-sectional surveys in Uganda (46). In a qualitative study conducted in Uganda, the absence of food to alleviate the side effects of Praziquantel was found to be one of the reasons causing low uptake (47).

#### Limitations of the study

One obvious limitation in this study is the recall bias given the fact that the MDA with PZQ for schistosomiasis in Ng'ombe township took place in 2018, and at least almost three years had passed by the time this study was conducted. Therefore, to manage this bias, the questionnaires used were not only well-designed and specific, they were also pretested, collected using the commcare application, and administered by trained research assistants.

The other limitation of the present study pertains to the impact of the Coronavirus (SARS-Cov-2) pandemic, which prevented traditional survey conduct in Ng'ombe township. Therefore, the survey was conducted over the phone to prevent transmission of the virus between the researcher, research assistants, and study participants. Additionally, phones were more time-efficient and accounted for the limited resources available. Moreover, given the nature of the survey conducted in the COVID-19 plagued-city, only adults were included in the study.

Despite the above limitations, the findings in the present paper are pertinent to the success of futures MDAs and the elimination of schistosomiasis, as almost identical results were found in similar settings either in terms of the coverage of the previous MDA or in terms of the predictors of the uptake of the MDA for Praziquantel.

## Conclusion

The findings in this study showed a lower coverage of MDA with PZQ than the target recommended by the WHO. This suggests more effort is needed to reach groups of people that have been less represented in urban populations to attain universal health coverage. The finding that prior knowledge of the MDA intervention and the use of incentives, such as snacks, during the MDA were important for the uptake of the MDA with PZQ suggests there is a need to enhance community engagement and intervention with effective communication strategies, particularly before the implementation of an intervention, to achieve targeted coverage. The integration of economic empowerment strategies in an economically vulnerable community such as Ng'ombe township is crucial for community responsiveness to health interventions, such as MDA with PZQ.

Future large studies may help provide a clear understanding of the role age, marital status, and employment status play as negative predictors of the uptake of the MDA with PZQ. Exploring barriers and enablers to implementation of the MDA with PZQ using qualitative approaches may also provide an in-depth understanding of the role of some factors in the uptake of PZQ in an urban slum setting of Lusaka.

## Data Availability

The raw data supporting the conclusions of this article will be made available by the authors, without undue reservation.
